# Supplementary material for: Are carbon emissions trading and green financial instruments synergistic? -Comprehensive quantitative research based on content analysis
Source: PLoS One. 2024 Mar 7;19(3):e0298601. doi: 10.1371/journal.pone.0298601 (PMC10919601; doi:10.1371/journal.pone.0298601)
Supplement: S1 Data — (ZIP) [file pone.0298601.s001.zip › Data/地级市Z_F工作报告.zip!/╣π╢1⁄2/╒╪╟∞╩╨/└┤╫╘╣1⁄2╓┌║┼í╛┬φ┐╦╩2╛▌═°í┐_2011.docx]

各位代表：

我代表肇庆市人民政府向大会作工作报告，请予审议。并请政协委员和其他列席人员提出意见。

2010年发展成效显著 “十一五”规划目标圆满实现

过去一年，是我市经济社会发展取得显著成效的一年。在省委、省政府和市委的正确领导下，在市人大及其常委会和市政协的监督支持下，市政府深入贯彻落实科学发展观，加快转变经济发展方式，全面实施《珠江三角洲地区改革发展规划纲要》（以下简称《规划纲要》），解放思想，迎难而上，开拓进取，争先进位，全力以赴促发展，坚定不移调结构，扎扎实实惠民生，推动全市经济社会平稳较快发展，全面完成或超额完成了市十一届人大五次会议确定的目标任务。全市呈现出工业经济提速增效，农村经济稳步增长，第三产业繁荣兴旺，城乡区域同步发展，社会事业全面进步，人民生活不断改善的良好态势。突出体现在以下十个方面：

——GDP突破千亿大关。围绕尽快实现GDP超千亿元的目标要求，全力推动经济加快发展，各项主要经济指标增速居全省和珠三角前列，实现了GDP超千亿元的目标。完成本地生产总值1065.9 亿元，比上年增长(下称增长)17.1% ，超过预期目标3.1个百分点，比全省平均增幅高4.9 个百分点。

——财政收入争先进位。全市地方财政一般预算收入达76.8亿元，增长37.36%，超过预期目标22.36个百分点,比全省平均增幅高13.61个百分点，增速居全省第二位，总量跃居全省第九位。各县（市、区）地方财政一般预算收入呈现良好增长态势，肇庆高新区、四会市和四个山区县增幅均超40%，高要市、四会市总量分别突破13亿元和11亿元，肇庆高新区和端州区均超8亿元。鼎湖区税收收入占财政一般预算收入比重达82.8%，为全市比例最高。

——产业结构不断优化。一、二、三产业比例从上年的19.2∶37.1∶43.7变为17∶42.6∶40.4，在2010年度珠三角产业竞争力排行榜中，我市的产业结构升级排名第三。工业主导地位逐渐形成，规模以上工业实现增加值408.15亿元，增长33.7%，超过预期目标11个百分点，增幅居全省第三位。第三产业繁荣兴旺，实现社会消费品零售总额332.9亿元，进出口贸易额43.9亿美元，分别增长21.1%和34.5%；商品房销售额、销售面积分别增长46.1%和22.9%；主要旅游景区（点）接待游客2008.5万人次，旅游收入109.23亿元，分别增长25.3%和37.5%。农业产业化水平提高，实现农林牧渔业增加值181.29亿元，增长6.3%；珍贵树种种植面积、柑桔产业产值居全省首位，集体林权制度改革走在全省前列。

——大旺园区成功升级。肇庆高新区经国务院批准升级为国家高新区，被人民网评为中国十大经济活力开发区，荣获省“双转移”特别贡献奖，一批重大产业项目相继落户，亚洲铝业二期建成投产，国电大旺热电联产、中恒生物医药项目正式动工，全区实现规模以上工业总产值361.83亿元，增长41.1%。以肇庆高新区为龙头的各类产业园区迅速崛起，园区的开发和招商引资成效显著。在省产业转移目标责任制考核中，我市“双转移”工作及中山（肇庆大旺）产业转移工业园均获优秀等次。

——广贺高速顺利通车。广贺高速（怀集至三水段）建成通车，结束了我市山区县没有高速公路的历史，大大缩短了与珠三角核心区的距离。同时，321国道小湘至马房段升级改造全面完成，珠外环高速高要至江门段、广肇高速二期建成通车，贵广、南广铁路肇庆段建设加快推进，珠外环高速肇庆至花都段、二广高速怀集至连州段动工建设，广佛肇高速三水至小湘段、怀（集）罗（定）高速等项目前期工作全面启动。全市重点建设项目122项，完成投资244.1亿元，其中列入省重点项目49项。全社会固定资产投资完成625.21亿元，增长35.1%。

——招商引资成果丰硕。第八届“肇庆金秋”经贸洽谈会签约项目39宗，投资总额406亿元。全年实际吸收外资9.34亿美元，增长5.2%，增幅高于全省平均水平。成功引进投资总额131.9亿元的中恒（大旺）国际生物医药物流产业基地。“双转移”成效显著，引进产业转移项目202个，投资总额444.13亿元；培训农村劳动力3.5万人，转移就业5.09万人。

——县域经济加快发展。县域（两市四县）生产总值、地方财政一般预算收入、全社会固定资产投资分别占了全市总额的64.3%、57.7%、61.4%，综合实力明显提升。四会市GDP超150亿元，有4家企业列入首批广东省现代产业500强，县域经济综合发展力居全省前三强。高要市GDP超200亿元，产值超亿元工业企业达104家，引进一批投资超10亿元大项目。广宁县实际吸收外资总量列四个山区县首位，特色经济集聚效应突出，成为“省县共建林浆纸一体化产业技术创新示范基地”。德庆县GDP增幅和工业增加值总量居四个山区县首位，农民人均纯收入增幅居全市前列。封开县华润水泥项目首期2条生产线达标达产，第3、4条生产线正式投产，全年上缴税收1.5亿元。怀集县财政综合增长率跃居全省第二，固定资产投资总量和增幅位居四个山区县第一，GDP突破百亿元大关。

——宜居城乡日新月异。“城市建设年”24项重点项目扎实推进，星湖大道、信安路改造工程和波海公园、牌坊广场、东门广场、砚都大道、肇庆大道等重点市政项目主体工程基本完成，修建绿道500公里，省立绿道105公里建设任务全面完成。启动“三旧”改造项目22个，动工面积2833亩，完成投资36.82亿元，端州龟顶新城示范性工程全面铺开。完成省下达节能减排目标任务，单位工业增加值能耗下降7.65%，单位生产总值能耗下降2.4%，被评为省节能先进市。8个中心镇污水处理厂全部建成。完成省下达削减化学需氧量和二氧化硫排放量目标任务，大气、水体等环境质量稳定达标。被国家住建部授予“中国人居环境范例奖”。

——社会事业全面进步。全年城镇居民人均可支配收入16832元，增长11.8%；农民人均纯收入7524元，增长19.6%，增幅创历史新高。年末城镇登记失业率为2.3%，创建国家级创业型城市通过省考评。新建教育强镇28个，为全省新创建最多的地级市；建成全省首个市职教公共实训中心，中、高等职业教育在校生规模均居全省前列。制定实施《肇庆市建设文化强市规划大纲》，端砚入选“岭南文化十大名片”。社会保险五大险种实现市级统筹，基本解决关闭破产企业退休人员和困难企业职工的参保问题。37.41万农村居民参加社会养老保险。城乡低保、五保对象实现应保尽保。新医改五项重点改革扎实推进，普通门诊纳入医保统筹，职工医保年支付限额提高到12万元。新型农村合作医疗实现农村常住人口全覆盖，年住院报销封顶线提高到6万元。市紧急医疗救援指挥中心和市疾控中心新大楼投入使用。实现村村有规范化卫生站，创建省卫生村437个，数量全省最多。有34人（次）获第十六届广州亚运会金牌。综治信访维稳三级工作平台建设获省一等奖。扶贫开发“双到”工作走在全省前列，全市已投入帮扶资金2.36亿元，已有149个贫困村、1.6万户贫困户、6.24万人实现脱贫。

——惠民大事扎实推进。广佛肇高速公路小湘至德庆、封开段可行性报告已上报省交通运输厅；完成29个农贸市场升级改造；牌坊广场改造扩建和环星湖栈道工程已完成并向市民开放；市第一人民医院新院、市图书馆新馆建设完成三分之二主体工程，卫生村和生态文明村创建任务超额完成；正在积极采取措施缓解端州城区交通拥堵问题；百花中学主体工程已经封顶，市一中新校区建设加快推进；七星岩东门广场已建成开放；全市新建廉租住房278套、经济适用住房346套；景丰联围除险加固工程主体建设任务基本完成；农村饮水安全工程超额完成，解决19.33万人饮水不安全问题。据国家统计局肇庆调查队调查结果显示，市民对市政府2010年实施为民惠民十件大事的总体评价满意率达98.3%。

各位代表，过去一年全市经济社会发展成效显著，为“十一五”发展划上了圆满的句号。五年来，全市人民紧紧围绕建设繁荣活力、文明法治、和谐安康、生态环保肇庆的奋斗目标，以科学发展为主题，抢抓机遇，攻坚破难，真抓实干，圆满完成了“十一五”规划各项目标任务，在科学发展、加快发展、和谐发展的轨道上迈出了可喜步伐。

“十一五”期间，我们大力发展四大主体经济，综合实力不断提升。经济规模不断壮大，五年本地生产总值翻了一番多，年均增长16.2%。地方财政一般预算收入增长2.76倍，年均增长30.3%。五年新增规模以上工业企业421家，规模以上工业增加值增长3.8倍。已有农业龙头企业111家,农林牧渔业增加值增长27.9%。五年间社会消费品零售总额和进出口贸易额分别增长1.3倍和1倍，主要旅游景区（点）接待游客和旅游收入分别增长1.5倍和1.7倍，商品房销售面积和销售额分别增长2.2倍和5.6倍。投资规模不断扩大，累计完成全社会固定资产投资1950亿元，比“十五”时期增长2.1倍。科技创新能力不断增强，全社会研发费用五年增长4.2倍，新认定高新技术企业61家，累计获授权专利1800多件，获得省级以上科技进步奖28项，风华高科获评“广东十大创新企业”。我市荣获“中国城市信息化创新奖”和“中国农村信息化领先地区”称号。金融服务水平不断提高，2010年末金融机构本外币存款余额1072.50亿元，贷款余额652.01亿元，五年分别增长1.2倍和1.8倍。民营经济发展加快，2010年实现规模以上民营工业总产值816.34亿元，个体私营企业实现税收55.69亿元，五年分别增长近4倍和6.4倍。

“十一五”期间，我们抢抓实施《规划纲要》重大机遇，一体化发展逐步加快。完成实施《规划纲要》“一年开好局”目标任务，主动参与省珠三角一体化规划编制，“五个一体化”专项规划和“四年大发展”工作方案正式实施，实现与珠三角城市异地缴存住房公积金贷款，公交等基本公共服务设施互通系统逐步推行。广佛肇经济圈合作加强，与广州、佛山共同签署经济建设合作框架协议和系列专项合作协议，建立市长联席会议制度，编制了《广佛肇经济圈发展规划》，首批37个重点合作项目正在推进，第二批重点合作项目基本确定，广佛肇城际轨道等项目按计划推进。大力加强产业招商合作，我市承接产业转移项目近八成来自广佛地区。车辆通行费年票互通互认，旅游、医保、教育、就业服务等合作全面推进。端州区与海珠区、高要市与高明区、四会市与三水区、怀集县与南海区建立起友好共建关系。

“十一五”期间，我们全力推进城乡协调发展，区域竞争力明显增强。中心区、山区生产总值年均分别增长17%和14.4%，城镇居民人均可支配收入和农民人均纯收入五年分别增长67%和69%。中心区成为推动全市经济加快发展的重要引擎。端州区大力发展现代服务业，人均GDP和人均财政一般预算收入等指标居全市第一。鼎湖区加快交通基础设施等重大项目建设，广新工业园、中储粮物流等大项目相继落户。四会市综合竞争力不断提升，五年内有三年财政综合增长率居全省前列。高要市财政综合增长率2007、2008年连续排名全省第一，生产总值和地方财政一般预算收入居全市首位。山区县成为全市经济发展的新增长点。广宁县林浆纸一体化、再生资源等支柱产业发展加快，2010年财政综合增长率跃居全省第九。德庆县推动工业产业集聚发展，现代农业发展壮大，新农村建设走在全省前列。封开县重大项目谋划建设成效突出，华润水泥项目累计完成投资42.3亿元，实现财政综合增长率从2007年全省末位到2009年排名第一的飞跃。怀集县矿产资源、林木加工、水电产业规模壮大，五年间地方财政一般预算收入增长2.6倍。

“十一五”期间，我们狠抓招商引资和重大项目谋划建设，发展后劲持续增强。产业园区建设迈上新水平，大旺园区成功竞得首批省示范性产业转移园5亿元专项扶持资金，五年投入园区建设资金超100亿元，实际吸收外资15.1亿美元，引进企业超过500家。全市建成3个省级产业转移工业园、2个省级产业集群升级示范区。招商引资成效显著，新引进一批世界500强企业，华润集团、中国国电、北新建材、广新集团、广晟集团、中恒集团、光大集团等大型国企民企投资落户。五年实际吸收外资41.1亿美元，比“十五”时期增长1.5倍。2008年以来，全市共引进产业转移项目617个，计划总投资934.48亿元。重大项目谋划建设加快推进，重点项目五年累计完成投资973亿元。新增高速公路148.4公里、等级公路2501公里。肇庆新港码头正式对外通关开放。

“十一五”期间，我们不断推进宜居城市建设，城乡面貌焕然一新。修编完成《肇庆市城市总体规划》，中心城区规划面积扩大到194平方公里。完成城东新区基础设施建设一二期工程。城镇化水平提高到45.6%。创建省卫生镇5个、省卫生村1910个和省级生态示范镇2个、生态文明村3144个。全面实现“十一五”节能减排目标，单位GDP能耗和化学需氧量分别下降16.5％和9.14%。圆满完成省下达淘汰落后产能任务。肇庆高新区被列为省产业转移循环经济工业园区。全市单位建设用地二、三产业增加值期末比期初增长1.4倍。实现耕地占补平衡，期末耕地保有量266.76万亩。新建污水处理厂18座，实现“一县一厂”目标，城镇污水集中处理率由20%提高到70%。全面完成林业生态县创建工作，森林覆盖率达67.7%。获得“国家环境保护模范城市”和“省绿色照明示范城市”称号。

“十一五”期间，我们努力深化重点领域和关键环节改革，体制机制不断完善。市、县两级政府机构改革顺利完成，县级大部制改革试点工作扎实推进；完成市政府第三、第四轮行政审批制度改革；开展简政强镇事权改革，市政府向各县（市、区）下放行政管理事项197项。事业单位分类改革和人事制度改革有序开展。端州区完成“撤镇设街”工作，“村改居”试点稳步推进。实施端州城区环卫管理体制改革效果良好。鼎湖区管理体制进一步理顺。公共财政体系不断完善，省直管县财政体制改革封开试点工作进展顺利。实施市属政府投资项目代建制。国企改革不断深化，市属国有企业由432家整合为83家，蓝带啤酒、风华集团和星湖科技完成重组并成功引入战略投资者。新增2家企业在A股上市。城信社退市、农信社改革取得阶段性成果。农村土地承包经营权流转进一步规范。集体林权制度改革主体任务全面完成，四会市试点经验在全省推广。医药卫生体制改革、文化体制改革扎实推进。实施农民工积分入户制度，有7579名符合条件的农民工入户城镇。

“十一五”期间，我们加快发展以五大民生工程为重点的社会事业，和谐肇庆建设取得新进展。创建教育强市扎实推进，全面实行免费义务教育，初中三年巩固率排全省第二，提前两年普及高中阶段教育，乡镇中心幼儿园开办率达100%，提前5年实现省定普及15年教育目标。职业技术教育发展速度和规模居全省前列，建成３所“万人职业技术院校”。县（市、区）100%实现中小学教师工资福利待遇“两相当”，符合条件的代课教师100%转为公办教师。文化强市建设力度加大，建成各级公共文化场馆1730个，“农家书屋”775个。列入国家级非物质文化遗产保护项目1项、省级15项。全民健身工作扎实推进，竞技体育屡创佳绩。医疗卫生事业发展加快，公共卫生服务体系进一步完善，重大传染病得到有效防控。覆盖城乡的社会保障体系初步建立，五大社会保险扩面征缴任务全面完成。累计培训农村劳动力17.51万人，实现转移就业27.51万人，城镇新增就业人数21.47万人。多渠道建设保障性住房4540套，完成农村危房改造11693户。完成农村饮水安全工程61宗，帮助50.21万人解决饮水不安全问题。水库移民安居工程完成投资9842万元， 2726户搬进新居。完成农村公路硬底化3406公里，具备通车条件的行政村班车通达率达100%。累计投入资金4亿多元，救助灾民80多万人次，帮助9504户受灾家庭重建家园。地质灾害防治投入资金1.07亿元，治理隐患点370处，搬迁1073户。对口援建汶川县克枯乡工作任务全面完成。安全生产形势稳定。应急管理体系不断完善。综治维稳基础不断夯实，连续三届获得“全国社会治安综合治理优秀地市”称号，荣获全国综治“长安杯”，公众安全感居全省前列。完成农业普查、经济普查、人口普查等重大统计任务。国防建设和民兵预备役工作得到加强，顺利完成第二批基层民兵营（连）“四个基本”建设达标任务。民族、宗教、外事、侨务、港澳、妇女儿童、老龄、残疾人、红十字、档案、人防、地方志、气象、地震等工作取得新成绩。

“十一五”期间，我们着力打造为民务实廉洁高效政府，行政效能不断提高。自觉接受市人大及其常委会的监督和市政协的民主监督，主动向市人大常委会报告和向市政协通报政府工作，组织办理人大代表意见批评建议502件、政协提案881件，答复率为100%，代表和委员均表示满意或基本满意。行政执法监督和行政复议工作不断加强。重大事项社会公示和听证制度得到落实。认真听取各民主党派、人民团体、工商联、无党派人士及专家顾问对政府重大决策的意见建议，网络问政有序开展。全面完成“五五”普法工作任务，法治城市、法治县（市、区）和“民主法治示范村（社区）”创建工作深入推进。全面推行政务公开。实行领导干部定期接访制度。加强效能监察，实施行政问责制和倒逼机制。市政府门户网站连续多年获得国家级奖励，建立“一站式”电子政务服务系统和行政审批电子监察系统，实现网上并联审批。落实党风廉政建设责任制，行政监察、审计监督扎实有效，“行风热线”越办越好。“打假”、 “打私”、“打非”工作扎实有效。国税、地税、海关、边检、海事、检验检疫、工商、质量监督等中央、省驻肇单位和金融机构为支持地方发展作出了积极贡献。

各位代表，过去五年，是我市经济发展最快、城乡变化最大、人民受益最多的五年，我们既成功应对了国际金融危机的严重冲击，又紧紧把握住从国家战略层面整体纳入珠三角一体化发展的重大机遇，发展历程令人难忘，发展成就令人振奋。这些成绩的取得，是省委、省政府和市委正确领导的结果，是市人大、市政协监督支持的结果，凝聚着全市上下、社会各界的智慧和汗水。在此，我代表市人民政府，向全市工人、农民、干部职工、外来务工人员、驻肇单位、驻肇部队、武警官兵以及各界人士致以崇高的敬意！向关心和支持我市建设和发展的外来投资者、港澳台同胞、海外侨胞和国际友人表示衷心的感谢！

我们也清醒地认识到，我市经济社会发展还存在不少困难和问题：一是经济总量不大、质量有待提高，重大项目特别是辐射带动能力强的重大工业生产性项目不多，自主创新能力有待增强；二是节能减排压力较大，节约集约用地水平有待提高；三是通胀预期压力加大，企业生产经营成本上升；四是改善民生任务依然繁重，社会保障水平有待提高；五是政府职能转变仍未完全到位，机关作风建设有待加强。我们要高度重视面临的困难和问题，采取切实有效措施认真加以解决。

科学谋划“十二五”发展 推动经济社会实现新跨越

“十二五”时期，是我市加快转变经济发展方式的攻坚时期，也是加快融入珠三角一体化发展、尽快建设成为名副其实珠三角城市的关键时期。综观国内外形势，我市既面临重大发展机遇，又面临着不少挑战，总体上正处于实现争先进位、跨越发展的战略机遇期。经济全球化深入发展，世界经济逐步恢复性增长，国际环境总体有利于我国和平发展。我国经济发展长期向好趋势不变，经济结构转型加快，内需市场潜力巨大。省委、省政府作出“加快转型升级，建设幸福广东”的部署，全面实施《规划纲要》，形成了新一轮发展热潮。广佛肇经济圈建设加快推进，为我市充分发挥后发优势，实现科学发展新跨越提供了强大动力。改革开放以来，我市干部群众逐步形成解放思想、团结一致、奋发图强、争先进位的强烈愿望和良好氛围。特别是经过了“十一五”时期的快速发展，我市经济总量不断壮大，综合竞争力大幅提升，发展后劲明显增强，为“十二五”打下了坚实的基础。

但与此同时，当前影响经济增长的不确定因素仍然很多，全球经济复苏动力不强，进程仍将艰难曲折，贸易保护主义不断加剧；随着国家宏观政策的调控，用地、用电、融资、节能减排等方面的压力加大；我市与珠三角核心区发展落差仍然很大，兄弟市发展迅猛、百舸争流，在你追我赶的竞争态势下，我们面临着做大经济总量与提升发展质量的双重任务，面临着实现经济转型升级与提高人民群众幸福感的双重责任，面临着加快经济发展与保护生态环境的双重要求。进入“十二五”时期，我们必须按照市委十届十次全会的决策部署，抢抓机遇，应对挑战，锐意进取，加快转型升级，敢于争先进位，努力开创“十二五”科学发展新局面。

“十二五”时期政府工作的总体要求是：高举中国特色社会主义伟大旗帜，以邓小平理论和“三个代表”重要思想为指导，深入贯彻落实科学发展观，以科学发展为主题，以加快转变经济发展方式为主线，抓住富民强市、建设幸福肇庆这个核心，解放思想、改革创新、争先进位，全面落实《规划纲要》，积极实施工业强市、一体化发展、龙头带动、科教兴市和人才强市、绿色城市“五大战略”，努力建设活力肇庆、法治肇庆、文化肇庆、生态肇庆、幸福肇庆，为建设成为名副其实的珠三角城市、成为未来广东发展的新增长极、成为能够代表广东科学发展成果的城市打下更加坚实的基础。

“十二五”时期我市经济社会发展主要目标是：全市生产总值按现行统计方式年均增长14%以上，GDP总量比“十一五”期末接近翻一番，人均GDP年均增长13.3%，规模以上工业增加值年均增长20%以上，第三产业增加值占GDP比重提高到47%左右，地方财政一般预算收入年均增长15%，全社会固定资产投资年均增长20%以上，社会消费品零售总额年均增长14.6%以上。万元GDP能源消耗年均下降2.09%以上，全面完成省下达的主要污染物减排目标。城镇居民人均可支配收入年均增长10%，农民人均纯收入增长10%以上，期末城镇登记失业率控制在2.6%以内。

根据上述总体要求，要实现“十二五”规划目标，必须紧扣主题主线，紧紧围绕“推进富民强市、建设幸福肇庆”的核心要求，正确处理好发展质量与速度的关系、经济发展与注重民生的关系、率先发展与协调发展的关系、经济建设与生态建设的关系、改革发展与社会稳定的关系，扎实推进“六个着力”：一是着力发展工业经济，迅速壮大经济实力。坚持走制造与创造相促进、信息化与工业化相融合的新型工业化道路，以园区为载体，以发展壮大主导产业集群为重点，形成以工业为主导的经济结构，增强经济规模实力。二是着力实施《规划纲要》，加快一体化发展。全面对接珠三角，全力打造广佛肇经济圈，把中心区打造成为经济发展先导区域，带动山区全面融入珠三角一体化发展。三是着力实施龙头带动，推动经济跨越发展。大力打造区域发展龙头、园区发展龙头、产业发展龙头和企业发展龙头，以龙头带动经济超常规发展。四是着力抓好科教兴市和人才强市，提高自主创新能力。加强产学研合作，推进人才强市、文化强市、教育强市建设，大力培育和引进人才，不断提升发展软实力。五是着力加强节能减排和生态环保，积极打造绿色城市。加大生态环境保护力度，切实强化节能减排和污染整治，推动产业转型升级，加快建设宜居城乡，努力实现可持续发展。六是着力发展民生事业，加快建设幸福肇庆。全面推进社会民生事业发展，不断提高基本公共服务均等化水平，促进社会公平正义，共建共享幸福肇庆。

各位代表，我市“十二五”发展的宏伟蓝图已经绘就，科学发展、加快发展的春天已经到来。只要全市人民齐心协力、开拓进取，就一定能够开创幸福肇庆的美好明天！

扎实做好2011年工作 实现“十二五”发展开门红

2011年是“十二五”开局之年、关键之年。今年政府工作的总体要求是：深入贯彻落实科学发展观，全面贯彻党的十七届五中全会、中央经济工作会议和省委十届八次全会精神，按照市委十届十次全会的部署，紧紧围绕富民强市、建设幸福肇庆这个核心，深入实施《规划纲要》，加快转变经济发展方式，加快重大项目谋划建设，加快壮大经济综合实力，加快区域城乡协调发展，加快宜居城乡建设，加快社会民生事业发展，努力为“十二五”科学发展开好局、起好步。

今年经济社会发展的主要预期目标是：生产总值增长14%，人均生产总值增长13.3%，一、二、三产业增加值分别增长4%、19%和14%以上，其中规模以上工业增加值增长20%；地方财政一般预算收入增长15%，全社会固定资产投资增长20%，社会消费品零售总额增长18%，实际吸收外资增长5%，外贸出口总额增长8%，城镇居民人均可支配收入增长10%，农民人均纯收入增长10%以上，居民消费价格涨幅控制在4%左右；人口出生率和自然增长率分别控制在13‰和7‰以内，城镇登记失业率控制在2.8%以内，全面完成省下达的单位生产总值能耗和主要污染物减排任务。

实现上述目标，必须抓好以下十方面工作：

一、加快转变经济发展方式，促进经济又好又快发展。加快调整优化产业结构，积极推动列入广东省现代产业500强项目发展壮大，努力培育我市工业50强、现代服务业30强，壮大龙头支柱产业。推进信息化与工业化深度融合，着力提升工业发展水平,发展壮大先进制造业和高新技术产业，推进省市共建汽车发动机零部件基地建设，积极谋划汽车整车制造、新能源电动车项目；抓好国家级电子信息产业基地、生物医药和健康产业基地建设，加快发展高端新型电子信息、高端装备制造、生物医药、半导体照明、新材料、新能源、节能环保等战略性新兴产业；改造提升传统优势产业，打造新型建材产业基地、小五金制造和林浆纸一体化省级产业集群、金属压铸产业集群升级示范区，推进传统优势产业转型升级集聚区建设。加快发展现代服务业，推进端州区生产性服务业基地、肇庆新港物流园区、肇庆高新区综合保税区、鼎湖华润建材物流和中储粮物流等项目规划建设；加快建设“国际化旅游休闲之都”和“广东省旅游综合改革示范区”，推进区域旅游合作和星湖景区经营管理体制改革，整合建设一批旅游精品，加快景区景点旅游示范基地创建，打造国际旅游目的地；推进端砚文化村建设，培育发展商务会展、信息服务、文化创意、金融服务、总部经济。积极扩大内需，推行企业工资集体协商制度，引导企业工资正常增长，增强居民即期消费能力；加快实施“万村千乡市场工程”、“双百市场工程”、“农超对接”试点和城乡农贸市场改造；加快规划建设五大（端砚、玉器、五金、建材、柑桔）专业市场；继续推动家电、摩托车下乡和以旧换新，大力实施国民旅游休闲计划，推进“节能产品惠民工程”，发展电子商务、连锁经营和现代物流配送、家庭医疗服务等新兴消费领域；组织企业参加“广货全国行”活动，继续协同办好广佛肇名优产品推介会。

二、大力发展现代农业，加快社会主义新农村建设。认真贯彻中央农村工作会议和中共中央、国务院《关于加快水利改革发展的决定》精神，全面落实强农惠农各项政策。加大“三农”投入，提高财政支出用于农业农村的比重。加强以水利为重点的农村基础设施建设，抓好标准农田和标准鱼塘建设，切实提高农业应对自然灾害能力。稳定粮食播种面积，提高粮食综合生产能力。大力发展现代农业，发展壮大农业龙头企业特别是列入省现代农业100强项目，扶持发展现代农业经营组织，加快怀集平原区现代农业示范园、高要国家级现代农业产业园、西江北岸柑桔优质水果产业带、珠三角城市鲜活农产品生产基地建设，推进农业规模化生产、产业化经营和社会化服务，做强做优农业主导产业。扩大珍贵树种种植规模，打造花卉、油茶产业化生产基地。加大优质农产品认证力度，健全农产品质量安全监管体系，加强农业科技创新和推广能力建设，做强农业品牌，提高特色农业竞争力。推进城乡基础设施一体化建设，提高城镇化水平。加快新农村建设，改善村镇人居环境，推进“岭南新民居”建设试点，创建省卫生镇10个以上，新增省卫生村受惠人口10万人。深化农村综合改革，巩固扩大集体林权制度改革成果，推进农村土地承包经营权有序流转，深化农村征地制度改革，推进农村集体经济制度化、规范化管理和公司化改革。全力推进扶贫开发“双到”工作，全面落实挂钩帮扶责任制，确保今年基本完成扶贫开发工作任务。

三、加快广佛肇经济圈建设，推动区域经济协调发展。加快实施“五个一体化”规划，全面落实“四年大发展”工作方案，推进“十大工程”项目建设。加强与广佛对接合作，认真落实三市市长联席会议议定事项，推动实施第一、二批重点合作计划项目，谋划共建广佛肇经济合作区。加快与广州铁路新客站、白云国际机场、南沙港对接的交通基础设施以及广佛肇高速公路三水至小湘段工程建设。建立健全区域环境污染联防联治机制，推动教育、医疗、卫生、社会治安和社会保障等公共事务管理深入合作。发挥中心区的区位交通、产业基础等优势，积极参与珠三角现代产业体系分工合作，着力建设现代产业集群，争取把肇庆高新区整体纳入省“十二五”规划的重点发展区域。发挥山区资源优势和生态环境等优势，加强沿江沿路产业园区规划建设，发展壮大新型建材、林产化工、矿产开发和综合利用、林木和厘竹深加工等特色产业，努力把资源优势转化为经济优势。

四、大力抓好招商引资，发展壮大园区经济。积极参加省组织的对央企、民企和世界500强招商活动，千方百计引进投资超50亿、超100亿元的重大生产性项目。发挥国家高新区的品牌效应，以创建省产业转移园典范和国内一流高新区为目标，提升肇庆高新区基础设施和配套设施功能，做大做强主导产业，加快建设现代工业城。抓好中山（肇庆大旺）园区扩园和中山大涌（怀集）、顺德龙江（德庆）园区规划调整申报，推动有基础的园区申报设立省级产业转移工业园，加快肇庆新港临港工业园、华润水泥新型建材产业园规划建设，积极承接产业转移。调整优化劳动力培训结构，积极参与全省“百校扶百县”活动，创建国家级和省级农村劳动力转移就业示范县。落实重大招商项目促建促产促效益制度，加强对亚洲铝业、华润水泥、亚洲金属资源再生工业基地等项目跟踪服务，力促项目增资扩产。放宽民营经济准入条件，进一步激活民间投资，促进民营经济加快发展。提高口岸服务水平。拓展对外贸易，推动外贸市场多元化发展，扩大具有自主知识产权、自主品牌和高附加值产品出口，促进加工贸易转型升级。

五、狠抓重大项目谋划建设，进一步增强发展后劲。推进中恒生物医药、封开铜钼矿开发、国电大旺热电联产一期、大唐国际高要热冷电联产等项目建设。继续争取肇庆核电厂、封开抽水蓄能电站等项目进入国家相关规划。加快端砚文化特色产业集聚园、玉器文化园、中巴软件园建设。配合建设广佛肇城际轨道肇庆段、贵广和南广铁路肇庆段，力争柳肇铁路肇庆段年内动工建设。加快肇庆火车站东站规划建设。推动广佛肇高速小湘至德庆、封开段立项建设。推进二广高速怀集至连州段、珠外环江肇高速二期建设，全面完成出省、市通道建设和县（市）之间国、省道改造任务。抓紧做好怀（集）罗（定）高速肇庆段、汕湛高速肇庆段前期工作。争取立项建设肇庆至白云国际机场快速干线，配合开展西江航道肇庆至封开界首段综合整治。加快电网、电源和配电工程改造建设。力争完成西气东输二期工程肇庆段、省天然气管网工程肇庆段建设。全面完成景丰联围除险加固达标工程。努力争取更多的重大项目列入国家和省的“十二五”规划。

六、积极实施“科教兴市”战略，努力提升自主创新能力。制定支持自主创新政策措施，加大财政扶持力度，引导企业和社会增加投入。发挥肇庆国家高新区龙头作用，建设科技创业园、科技创新中心和科技产业项目孵化器。再组织申报一批省级工程技术开发中心和高新技术企业，完成每百万人口30件专利技术的考核指标。支持企业与高校、科研院所建立产学研战略联盟，建设一批产学研结合示范基地。培育一批省级技术创新示范专业镇，加快建设星火技术产业带。实施人才强市战略，优化人才工作政策和环境，加大创新科研团队和创新创业带头人的引进力度，吸纳50名科技特派员进驻企业。大力实施品牌战略，积极争取更多产品获得省政府质量奖，保护注册商标，再创一批中国驰（著）名商标和名牌产品。加强科普知识教育，提高公民科学知识素质。着力建设教育强市，确保完成省教育强市（区）、教育强镇创建任务；高标准、高质量普及九年义务教育，巩固提高学前教育到高中阶段教育普及成果，推进义务教育均衡发展；加快职教基础建设，加强肇港澳职业技术教育合作，支持职教院校提升办学层次；加快乡镇中心幼儿园和村办幼儿园建设，鼓励扶持民办幼儿园发展；提高高等教育和特殊教育水平；建立专业化教师队伍，全面推进素质教育。

七、切实加强城乡建设管理，进一步提高宜居城乡建设水平。加快编制中心城区和县城控制性详细规划，抓好中心镇和村庄规划编制试点工作。做好城市发展战略性规划、区域功能布局规划、产业选择规划，继续开展“城市建设年”活动，切实加大城市管理力度。推进肇庆城区基础设施建设和西江三桥等“一江两岸”、“东扩南连”交通设施建设，积极推进市行政服务功能配套建设，加快建设完善牌坊广场、砚都大道、肇庆大道、大冲广场和东、西调洪湖等重点市政工程，加强星湖水质保护，努力打造“东方日内瓦”。加强端州城区公共交通建设，规划改造江滨堤路、西江路，推进端州路过街隧道建设，进一步缓解交通拥堵问题。加快“三旧”改造步伐，推进端州龟顶新城、城东城市综合体等重点项目建设。加快城区旅游配套设施建设，加强城市地下空间规划利用，扩大完善绿道网，加强绿道网的管理和维护。加大城区市容市貌整治力度，提高城区绿化覆盖率，进一步净化、绿化、美化、亮化城市。提升房地产建设管理水平，促进房地产业健康发展。加大节能预警调控力度，加快淘汰落后产能，加强重点耗能企业、公共机构节能监察，大力推广高效照明节能产品，鼓励企业实施节能技改，加强非工业领域尤其是建筑节能。落实污染减排问责制和一票否决制，强化环保执法监督，推进区域大气复合污染防治和河涌水环境综合整治，督促污水处理厂按期实现“一年60%、三年75%”处理量目标；加强饮用水源地环境监管和污染防治。确保通过国家卫生城市和环境保护模范城市的复查。认真落实耕地保护责任制，继续推进耕地开发补偿，实施差别化供地政策，加大闲置土地处置力度，开展节约集约用地水平量化考核。强化矿山储量动态监管和矿产资源开发督察。提升应对气候变化、防御气象灾害能力，加强地质灾害群测群防，确保人民群众生命财产安全。

八、继续创新体制机制，不断增强发展活力。推进重点领域和关键环节改革，深化行政审批制度改革，推进县级大部制改革。完善富县强镇、简政强镇事权改革和镇、村公共管理，加快端州区“村改居”工作。积极培植税源，支持税务部门强化税源监控和税收征管，规范非税收入征收管理，建立适应基本公共服务均等化要求的公共财政体系。认真落实省调整完善分税制财政管理体制实施方案，配合做好省直管县财政体制改革，完善财政预算编制执行管理、财政投资评审、政府采购和财政支出绩效评价制度，落实政府投资项目代建制。完善国资监管体制，健全国有资本经营预算和收益分享制度。继续推动企业上市发展，力争今年再有2-3家企业上市。推进农村信用社向农村商业银行有序改制，积极开展试点加快设立村镇银行。鼓励支持金融机构加大对重点建设项目、中小企业和“三农”的信贷扶持。吸引更多股份制商业银行落户我市。加快推进政策性水稻、农房等保险试点工作。注重防范地方性金融风险。大力推进医药卫生体制、文化体制改革和科技、教育、卫生、体育等事业单位分类改革。

九、加快发展各项社会事业，促进社会和谐稳定。繁荣发展文化事业，大力发展文化产业，完善市、县、乡镇（街道）、行政村（社区）四级公共文化设施网络，加快市图书馆新馆、市博物馆新馆、市文化馆、市文化艺术中心等重点公共文化设施规划建设，实施广播电视村村通、农家书屋、文化共享、农村电影放映全覆盖等文化惠民工程。加强公民道德素质教育，推进各项文明创建活动。加快市第一人民医院新院建设，力争四会、鼎湖、高要人民医院建设项目纳入中央、省级投资计划。积极发展中医药事业。着力保障和改善民生，多渠道开发就业岗位，落实和完善就业创业政策，加强劳动保障监察执法，保障劳动者合法权益。建设完善覆盖城乡居民的社会保障体系，推进新型社会养老保险，健全全民医保体系，提高社会保险待遇水平。加快推进低保救助标准城乡一体化，把年人均收入低于2200元的农村家庭纳入低保范围，建立城乡特困人员医疗救助制度。加大廉租房、公租房和经济适用房建设，完善住房保障体系。加强国防教育，推进基层民兵营（连）“四个基本”建设，力争今年全部达标，扎实开展拥军优属、拥政爱民，争创国家“双拥”模范城。切实办好市第九届运动会。加强和创新社会管理，提高社区建设水平，建立调处化解矛盾纠纷综合平台，抓好端州区深化社会管理体制改革试点。抓好各级敬老院的规划建设，市儿童福利院建成投入使用，推进社会福利和慈善事业发展。全面做好人口计生工作，稳定低生育水平。加强价格调控监管，切实稳定价格总体水平。加强食品药品安全监管。完善社会治安防控体系，打击各种违法犯罪活动。加大力度打击制假售假、走私贩私、非法传销。加强安全生产，健全安全生产事故预防预警和应急处置体系。创建全国综合减灾示范社区。继续抓好外事、侨务、民族、宗教、人防、地震、统计、档案、地方志、妇女儿童、老龄、残疾人、红十字、殡葬管理等各项工作。

　 十、加强民主法制建设，全面推进依法行政。自觉接受人大及其常委会的监督，严格执行各项决议决定，定期向人大及其常委会报告工作，积极支持人民政协履行政治协商、民主监督、参政议政职能，切实做好人大代表建议和政协提案办理工作。认真听取各民主党派、人民团体、工商联、无党派人士及专家顾问的意见建议。坚持科学民主决策，完善行政决策程序。积极推进网络问政，扩大公民有序参与。启动“六五”普法教育。加强效能监察，推进政务公开，健全倒逼机制，落实行政问责制，强化政府各部门的执行力。进一步精简会议、文件，严格控制各类评比达标、表彰庆典等活动，切实降低行政成本。继续办好“行风热线”。推进政府廉政建设和反腐倡廉各项工作，加强行政监察和审计监督，强化监督制约，规范权力运行。

各位代表，我们要学习“敢想、会干、为人民”的广州精神，把为人民群众办实事、谋幸福作为各项工作的出发点和落脚点，认真抓好市委十届十次全会确定的十项惠民工程的落实，继续推进市政府2010年为民惠民十件大事，抓紧完成广佛肇高速公路小湘至德庆、封开段立项建设和市第一人民医院新院、市图书馆新馆、市一中新校区、缓解端州城区交通拥堵等工程。在此基础上，市政府今年将继续集中力量办好十件惠民实事：一是向全市困难群众发放临时性价格补贴。建立和完善市价格调节基金，对困难群众实行临时性价格补贴。春节前市财政安排1080万多元专项资金，向全市108045名城乡低保对象、农村五保对象、享受国家抚恤补助的优抚对象、建国前入党的农村老党员每人一次性发放100元临时性价格补贴。二是扩大就业培训和社会就业。全市就业培训69000人，其中农村劳动力培训36000人，城镇新增劳动力及其他培训33000人；全市新增就业88400人，其中城镇新增就业45000人，农村培训转移就业40000人，大学生就业3400人。三是加快蔬菜基地建设。新建3个以上有一定规模的蔬菜基地，启动建设农副产品平价商店，严格控制物价过快上涨。四是加快推进城乡保障性安居工程。全市新建设城镇保障性住房6378套（户），农村危旧房改造1600户。五是加快教育强市建设。全市50%以上的镇创建成为省教育强镇，端州、鼎湖、四会、高要创建成为省教育强区（市）；端州城区分别新规划建设1—2所小学和优质幼儿园。六是启动西江三桥修建工程。加强“一江两岸”对接，加快城市交通基础设施建设。七是继续改善城区交通拥堵。加快端州路过街隧道建设，启动粤运客运站搬迁工作，进一步缓解城区交通拥堵。八是进一步完善景区配套设施。启动鼎湖山新入口规划建设，不断完善环星湖带19公里公共自行车休闲通道。九是改造美化城区出入口环境。实施城区出入口道路及周边环境的改造美化工程，各县（市、区）按属地化管理同步推进。十是切实加强城区主干道管理。落实城区端州路、天宁路、建设路、信安路、星湖大道、砚都大道等主干道交通、门前卫生“三包”、户外广告牌等严管措施，完成10块以上城区空地绿化，进一步绿化、亮化、美化城市环境。市政府将正式发文，明确牵头领导和责任单位，落实目标任务，健全督办机制，定期检查通报落实情况。

各位代表！回顾“十一五”，我们豪情满怀；展望“十二五”，我们信心百倍！加快科学发展、推进富民强市、建设幸福肇庆是时代赋予我们的光荣使命！让我们在中共肇庆市委的正确领导下，深入贯彻落实科学发展观，紧紧依靠全市人民，抢抓机遇，开拓创新，奋发有为，再创辉煌，为把肇庆建设成为名副其实的珠三角城市、成为未来广东发展的新增长极和能够代表广东科学发展成果的城市而努力奋斗！
